# H-type double gallbladder malformation: a case report

**DOI:** 10.1093/jscr/rjaf838

**Published:** 2025-10-21

**Authors:** Shaoyi Wang, Xiaozhou Yang, Sijia Feng, Qiangshan Bai, An Chen

**Affiliations:** Department of General Surgery, The Second Affiliated Hospital of the Air Force Medical University, Xi’an 710038, China; Department of General Surgery, The Second Affiliated Hospital of the Air Force Medical University, Xi’an 710038, China; Department of General Surgery, The Second Affiliated Hospital of the Air Force Medical University, Xi’an 710038, China; Department of General Surgery, The Second Affiliated Hospital of the Air Force Medical University, Xi’an 710038, China; Department of General Surgery, The Second Affiliated Hospital of the Air Force Medical University, Xi’an 710038, China

**Keywords:** gallbladder malformation, case report, double gallbladder

## Abstract

Biliary tract malformations are rare congenital anomalies, occurring in approximately 1 in 4000 individuals that may predispose to cholelithiasis and increase surgical risks, emphasizing the need for precise preoperative diagnosis. We report a 21-year-old male with recurrent biliary colic found to have a rare "H-type" duplex gallbladder during laparoscopic cholecystectomy—two separate gallbladders with independent cystic ducts. Pathology confirmed cholelithiasis with cholecystitis. The patient had an uncomplicated recovery with discharge on postoperative day 1. This case underscores the importance of recognizing such anatomical variants preoperatively to minimize surgical complications in biliary procedures.

## Introduction

Biliary tract malformations are relatively rare congenital anomalies, with double gallbladder malformations being even more uncommon, with an estimated incidence of 1 in 3800 to 5000 individuals [1]. Given that biliary variations increase surgical complexity and the risk of perioperative complications, it is essential to thoroughly evaluate the patient's gallbladder anatomy and prepare adequately for the perioperative period.

## Case presentation

**Figure 1:**
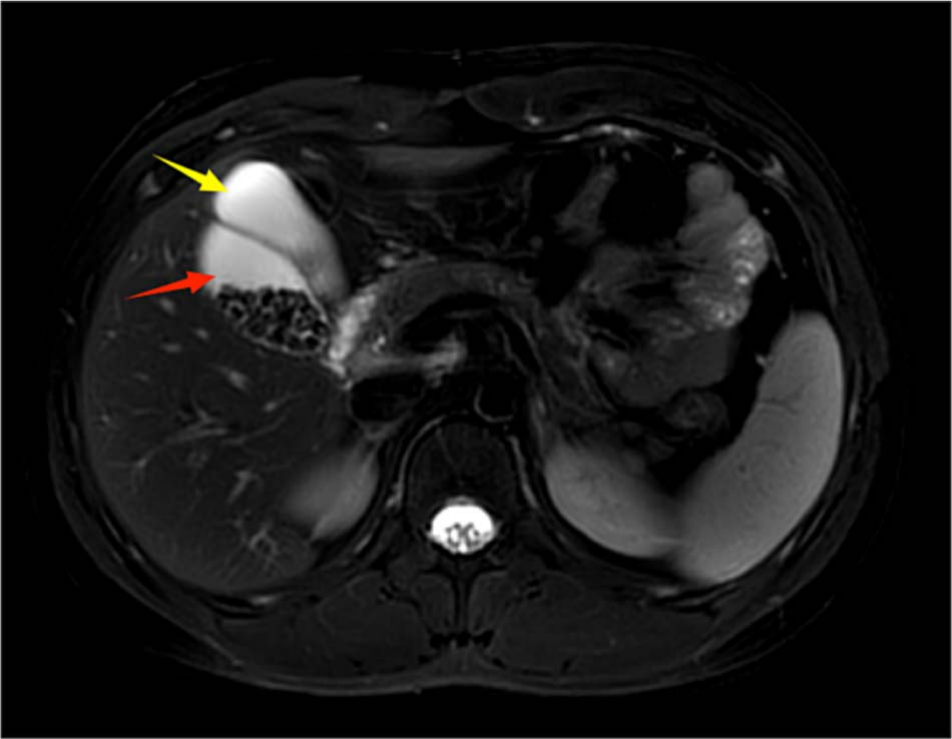
Upper abdominal MRI demonstrates a double gallbladder variation. The two arrows indicate the respective gallbladders, the lateral one of which contains multiple gallstones.

**Figure 2:**
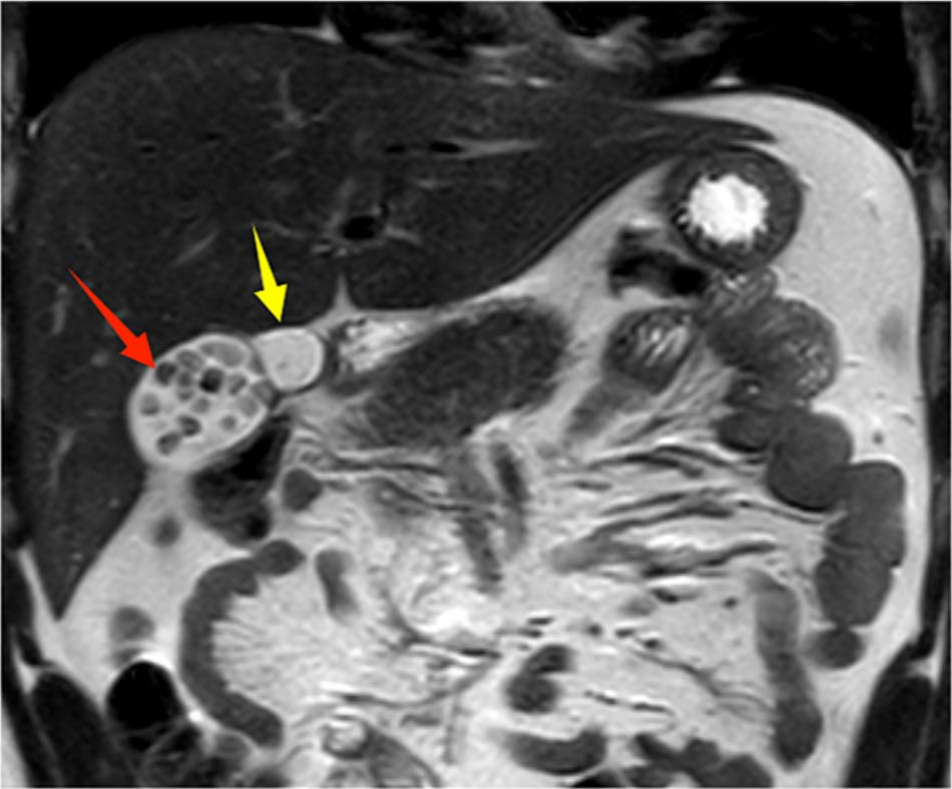
Upper abdominal MRI coronal view demonstrates a double gallbladder variation. The two arrows indicate the respective gallbladders, the lateral one of which contains multiple gallstones.

A 21-year-old male patient was admitted to our hospital with a 2-year history of intermittent right upper quadrant colicky pain. The symptoms initially occurred 2 years ago after consuming fried food, accompanied by nausea and vomiting, and were relieved after antispasmodic and analgesic treatment. Over the past 2 years, the symptoms recurred frequently, prompting the patient to seek further evaluation at our institution. The patient had no significant past medical history, including hypertension, diabetes, coronary heart disease, or other chronic conditions. He denied smoking, alcohol consumption, and any known drug allergies. Physical examination on admission revealed stable vital signs, with no jaundice observed in the skin or sclera. The abdomen was flat, with mild tenderness in the right upper quadrant and no rebound tenderness. The liver and spleen were not palpable. Murphy's sign was negative, abdominal percussion yielded tympanic sounds, and bowel sounds were normal. Laboratory tests, including complete blood count, liver function (bilirubin, ALT, AST, etc.), coagulation profile, amylase, lipase, and infection markers, were all within normal limits. Ultrasonography indicated multiple gallstones, a thickened and indistinct gallbladder wall, and suspicion of a folded gallbladder or possible double gallbladder malformation. To further delineate the biliary anatomy and ensure surgical safety, an abdominal MRI was performed. Magnetic resonance imaging (MRI) confirmed a double gallbladder malformation, with multiple stones in the lateral gallbladder (Figs 1 and 2). The clinical diagnosis was established as cholelithiasis with double gallbladder malformation.

**Figure 3:**
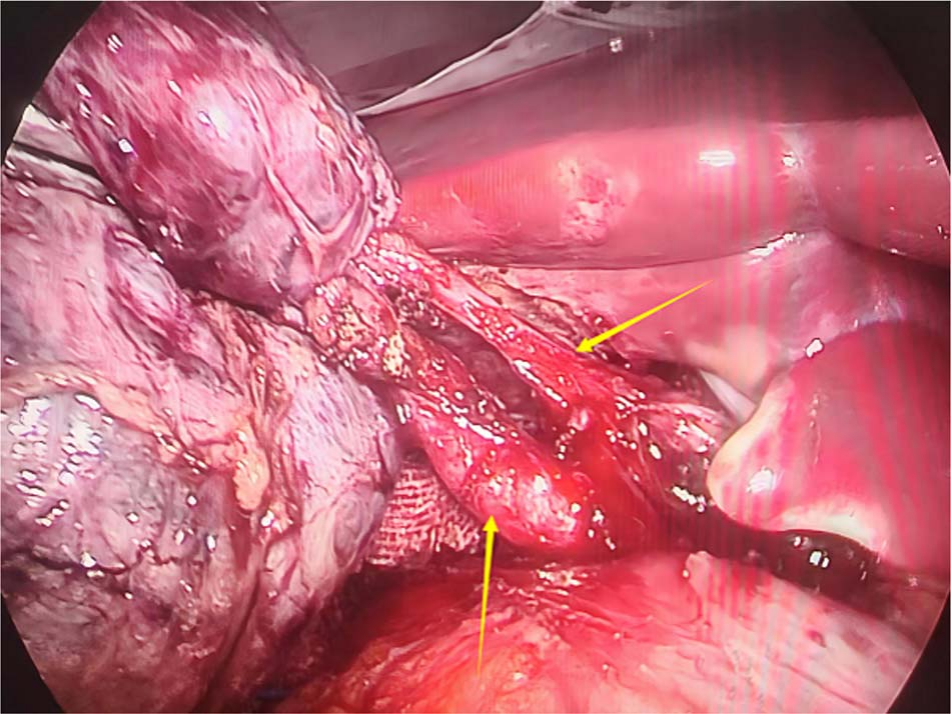
Intraoperative image showing two cystic ducts, each indicated by an arrow.

**Figure 4:**
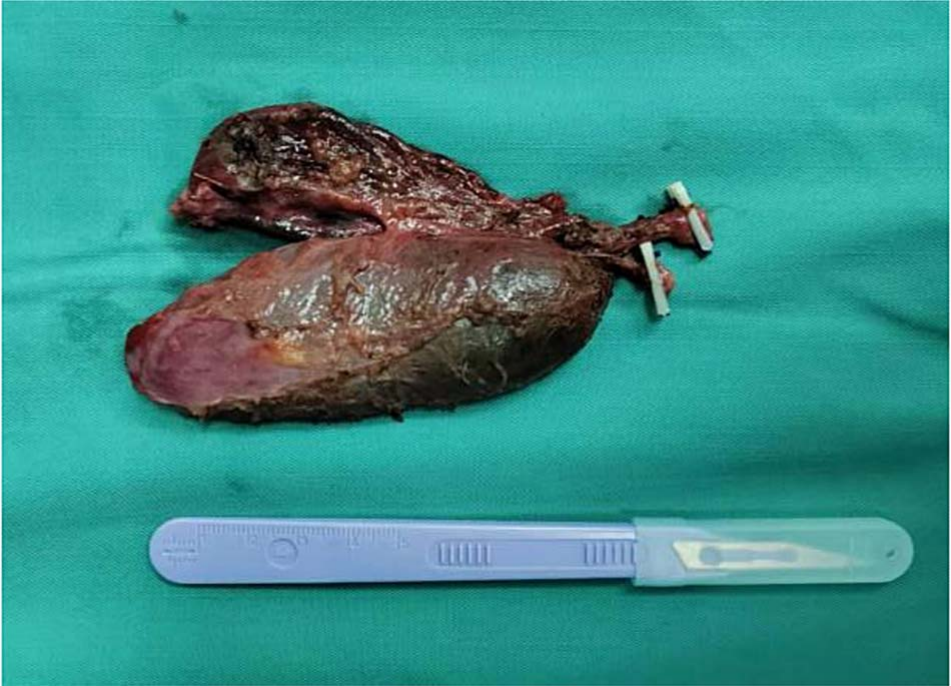
Postoperative specimen showing two completely independent gallbladders, each with its own separate cystic duct.

Given the patient’s recurrent biliary colic, laparoscopic cholecystectomy was decided. During surgery, adhesions between the gallbladder and the surrounding omentum were carefully dissected. After mobilizing the hepatic porta, the anatomy of Calot’s triangle was meticulously identified, confirming the double gallbladder malformation as preoperatively suspected. The two gallbladders had independent cystic ducts draining into the common hepatic duct (Fig. 3). The lateral gallbladder contained multiple stones, while the medial one was stone-free. A single cystic artery, originating from the right hepatic artery, was observed passing posteriorly between the two cystic ducts before supplying the gallbladders. The cystic ducts were sequentially clipped and transected using bioabsorbable clips. Postoperative specimen dissection revealed two gallbladders measuring 8.5 × 3.0 cm and 8.5 × 2.5 cm, respectively. The serosal surfaces of the two gallbladder bodies were adherent, and each had an independent cystic duct inserting into the common hepatic duct, confirming an "H-type" double gallbladder malformation (Fig. 4). Pathological examination reported a double gallbladder malformation: one with a slightly roughened mucosa, wall thickness of 0.2 cm, multiple stones, and features of adenomatous cholecystitis; the other with a smooth mucosa, wall thickness of 0.1 cm, and no significant stones. The patient recovered well and was discharged on the first postoperative day.

## Discussion

Double gallbladder malformation is an extremely rare anatomical variation, with a very low clinical incidence. It typically forms during the fourth week of embryonic development, either through epithelial organization of the choledochal cyst or abnormal differentiation of the primitive gallbladder [1, 2]. While gallbladder diseases are more common in females, double gallbladder malformation shows no significant gender predilection [3]. Boyden first described double gallbladder malformation and classified it into three types: Type I, a septated gallbladder with two chambers sharing a single cystic duct; Type II, also known as the Y-type, where two gallbladders are not connected internally, and their cystic ducts merge before draining into the common hepatic duct; and Type III, the H-type, where two gallbladders are not connected internally and each has its own cystic duct draining separately into the common hepatic duct [4]. Current diagnostic methods for double gallbladder malformation include ultrasound, computed tomography (CT), magnetic resonance cholangiopancreatography (MRCP), and endoscopic retrograde cholangiopancreatography (ERCP). MRCP is considered a safe and reliable option due to its lack of radiation, non-invasiveness, and high sensitivity [5]. Double gallbladder malformation may affect bile excretion and increase the risk of biliary diseases, including cholecystitis, cholelithiasis, and biliary obstruction. Asymptomatic patients generally do not require surgical intervention [6], but symptomatic patients are advised to undergo surgery as early as possible, typically involving the removal of both gallbladders [7, 8]. This approach not only prevents future complications such as cholecystitis, gallstones, or gallbladder cancer in the remaining gallbladder [9] but also reduces surgical difficulty and the risk of intraoperative bile duct injury [10–12]. Some researchers suggest that if a double gallbladder is discovered unexpectedly during surgery, conversion to open surgery may be necessary. However, with advancements in laparoscopic techniques, laparoscopic cholecystectomy is considered safe and reliable, whether the double gallbladder is identified preoperatively or intraoperatively [13, 14]. Nonetheless, it is crucial to carefully identify the cystic duct and cystic artery during the procedure.

## Conclusion

In conclusion, this case report serves as a reminder to surgeons that while cholecystectomy is a routine procedure, anatomical variations should not be overlooked. Thorough preoperative evaluation and careful surgical planning are essential to avoid unnecessary complications.

## Data Availability

All data available are included in this article and its supplementary material files. Further inquiries can be directed to the corresponding author.
